# Functional validation of the PSEN1 R358P and PSEN1 T119I variants in Alzheimer's Disease: an in vitro study

**DOI:** 10.1002/alz70855_098040

**Published:** 2025-12-23

**Authors:** Diego E. Garcia Chialva, Diego Cifarelli, Luciana Isaja, Manuela Apecetche, Laura Martínes Ojeda, Tatiana Itzcovich, Patricio Chrem Mendez, Gustavo Sevlever, Maria Scassa, Ezequiel Ignacio Surace, Leonardo Romorini

**Affiliations:** ^1^ FLENI‐CONICET, Escobar, Buenos Aires, Argentina; ^2^ FLENI, Escobar, Buenos Aires, Argentina; ^3^ FLENI, ESCOBAR, Buenos Aires, Argentina; ^4^ Fleni, Escobar, Buenos Aires, Argentina; ^5^ Fleni, Buenos Aires, Argentina; ^6^ Fleni, CABA, Buenos Aires, Argentina; ^7^ Fleni, Buenos Aires, Buenos Aires, Argentina

## Abstract

**Background:**

Alzheimer's disease (AD) is a neurodegenerative disorder and the leading cause of dementia worldwide. It is characterized by progressive neuronal degeneration and the accumulation of beta‐amyloid plaques (Aβ) and neurofibrillary tangles (NFT) in the brain. AD manifests in sporadic AD (sAD) and familial AD (fAD). fAD is associated with inherited genetic mutations affecting amyloid precursor protein (APP) processing, involving genes such as APP, PSEN1, and PSEN2.

**Method:**

The identification of two novel PSEN1 variants, p.T119I and p.R358P in early‐onset AD patients at FLENI provided a unique opportunity to study their possible implications in fAD. Notably, the patient harboring the PSEN1 R358P variant also carried a novel SORL1 variant (Gly1536Asp). Noteworthy, genetic variants in SORL1 are now considered a major AD risk factor. To evaluate the role of these two novel PSEN1 variants in APP processing, we developed a cellular model using PSEN1 Knock‐Out (KO) HEK293T cells created through CRISPR/Cas9 technology. We assessed the Aβ 42 /Aβ 40 ratio (AD biomarker) in the supernatant of PSEN1 KO‐cells transfected with expression vectors coding for APP and either wild‐type PSEN1, the novel PSEN1 variants or PSEN1 A246E (a known pathogenic mutation).

**Result:**

We observed a significant (*p* <0.05) increase in the Aβ 42 /Aβ 40 ratio in HEK293T cells transfected with PSEN1 A246E or PSEN1 R358P plasmids and a slight trend towards an increase in cells transfected with PSEN1 T119I vector. In the case of PSEN1 R358P‐transfected cells, the increase in the Aβ 42 /Aβ 40 ratio observed was primarily due to the decrease in Aβ 40 levels in the supernatant.

**Conclusion:**

These findings suggest a potential pathogenic role for the PSEN1 R358P variant in fAD, independent of the co‐occurring SORL1 mutation.

